# Immediate health and economic impact of the Tigray war on internally displaced persons and hosting households

**DOI:** 10.1038/s41598-023-45328-4

**Published:** 2023-10-23

**Authors:** Aregawi Gebreyesus, Afework Mulugeta, Abraha Woldemichael, Akeza Awealom Asgedom, Girmatsion Fisseha, Mache Tsadik, Tesfay Gebregzabher Gebrehiwot, Mengistu Mitiku, Molla Teferi, Hagos Godifay, Yibrah Alemayehu

**Affiliations:** 1 School of Public Health, College of Health Science, Mekelle University, P.O. Box 1871, Mekelle, Tigray Ethiopia; 2 Tigray Bureau of Health, Mekelle, Tigray Ethiopia

**Keywords:** Environmental social sciences, Health care

## Abstract

Globally, war is the major cause of displacement from the usual place of the biological environment. The war of Tigray exposed thousands of people to internal displacement and migration. Evidence has shown that displaced people and migrants shoulder the health and economic burden to ensure survival. However, evidence of the impact of the war on health and the economy related to the displaced people and their hosting communities is not documented. Thus, this study aimed to investigate the health and economic impact of the war on displaced people and the hosting community. A community-based survey was conducted among randomly selected 3572 households of 48 woredas/districts from August 06 to 30/2021 in Tigray. Each district had 4 enumeration sites and there were 20 households (HHs) to be sampled per each enumeration site. Data were collected using a pretested structured questionnaire using face-to-face interviews of displaced and hosting household heads. The entered data is exported to SPSS version 26 statistical packages for data analysis. Summary statistics and geo-spatial analysis was computed. The war had a significant impact on the health and economy of the community of Internally Displaced People (cIDPs) and hosting households. There were 12,691 cIDPs and 3572 hosting HHs. About 12.3% had chronic illness12.3% of (cIDP) who had chronic diseases and follow-up medication was forced to stop their medication. 536 (15%) civilian family members of cIDPs were killed at their homes. During the war, 244 (6.83%) of civilian family members faced physical disability. Consequentially, 43.8% and 58.8% of respondents of cIDPs suffered from severe depression and post-traumatic stress disorder. The war had a significant amount of personal resources such as domestic animals, cereals, cars, machinery, and HH furniture was looted and vandalized by the perpetrator forces from the cIDPs and hosting HHs. The range of family size in the hosting households was 3 to 22. The war had a significant health and economic impact on both cIDPs and hosting HHs. cIDPs suffered from various illnesses and disabilities related to the war with no medical access and follow-up care leading them to stressful situations such as depression and PTSD. There was also a huge economic damage and distraction which threatens the survival of the survivors.

## Introduction

War, one of the distressing events, has an enormous and tragic impact on public health and it accounts for more deaths and disabilities than many major diseases combined^1^. Yet, it has an overwhelming effect on a country's infrastructure, security, economic development as well as the mental and social well-being of its people (individuals, families, or entire communities). Besides, war may subject people to forced displacement, material loss, separation or death of relatives and friends, loss of control and autonomy, and lack of access to resources. The internally displaced people (IDP) may experience increased vulnerability and greater exposure to violence, poverty, lack of prospects, and uncertainty as to what is happening and what the future holds. Thus, people may feel overwhelmed, confused, frustrated, anxious, numb, and detached^2,3^.

The destruction of the health infrastructure leads to severely compromised health systems’ capacity to respond to the direct and indirect health consequences of the war. Based on the United Nations Higher Commissioner Refugees (UNHCR) April 2022 report shows that there are about 100 million displaced people; and from this, 53.2 million are internally displaced people (IDPs) from war conflicts and violence globally. The majority of the IDPs were from low and middle-income countries^4^.

The IDPs are one of the most pressing humanitarian problems and political issues faced by the global community^5^. Most IDPs are still living in low-income countries and half of them are in Africa living during war and persecution (United Nations Higher Commissioner Refugees [UNHCR]^6^. IDPs are those who have not crossed an internationally recognized state border. However, they are obliged to flee or to leave their homes or places of habitual residence, particularly to avoid the effects of armed conflict, situations of generalized violence, violations of human rights, and natural or human-made disasters^7^.

The Horn of Africa bore the brunt of displacement more broadly. UN reports and other studies revealed that displacements associated with ethnic conflict and violence in the Horn of Africa especially in Ethiopia and Somalia continue worsening^4,8–11^. During the past four decades, Ethiopia has been ravaged by large-scale civil war, conflict, and famine. As a result, a large number of people suffered traumatic experiences by being involved in ethnic conflict while very often losing homes, friends, and relatives^12^.

According to a current report from Internal Displacement Monitoring Center (IDMC) on global displacement, Ethiopia experienced the highest number of people fleeing their homes within the country in the first half of 2022^13^. The report added the current war in Tigray has intensified the problem. Since the conflict broke out in the Tigray region at the beginning of November 2020, civilian casualties from various zones of the region are victims of traumatic outcomes of the war^10,14^. Due to this conflict, numerous civilians have been forced to be displaced from their homes internally and externally. While many are displaced to neighboring countries, there has been an influx of internally displaced people (IDPs) into the big cities. The conflict in Tigray has affected almost every aspect of life and has caused widespread displacements resulting in 2.1 million Internally Displaced People (IDPs) found the whole of Tigray including the eight towns IDP centers; namely, Shire, Aksum, Adwa, Adi-grat, Wukro, Mekelle city, Mai-chew, and Abyi-adi^15,16^. Based on the Tigray social affairs bureau, 56% of the IDPs were hosted inside the community in each household as community IDPs.

Although stakeholders are struggling to bring immediate humanitarian support to the displaced community, the additional multi-sectorial effort is required for a better outcome. Internally displaced persons have a traumatic experience and are capable of provoking fear, helplessness, or horror in response to the threat of injury or death. Such traumatic events include lack of food, water, shelter, and medical care, imprisonment, combat and injury, abuse and isolation, torture, murder, and death of family members^17–21^. Several studies have shown that people who are exposed to such events are at increased risk for serious mental and Psychological disorders such as PTSD, major depression, panic disorder, generalized anxiety disorder, and substance abuse, as compared with those who have not experienced traumatic events^22^.

Different organizations and researchers have studied IDPs in Tigray mostly those found in IDP centers. However, the impact of the war on the health and economy of displaced people and their hosting communities is not well studied. Therefore, this study aimed to assess the health and economic impact of the war on community-hosted internally displaced persons and hosting households in Tigray.

## Methods

### Study area and setting

The study was conducted in all zones of Tigray except the western zone. The western zone was excluded as it was occupied by the invading forces. Data were collected from 48 woredas and 3840 households from August 06-30/2021. Tigray shares a border with Eritrea, North Sudan, the Amhara region of Ethiopia, and the Afar region of Ethiopia to the north, west, south, and east respectively.

### Study design and population

A community-based cross-sectional study was conducted among cIDPs and cIDP-hosting households during the eight-month war in Tigray (from November 4, 2020, to June 28/2021). Those internally displaced persons displaced during the war period and those hosted inside the community households were included in the study; means IDPs those were living in IDP camps are not included. We excluded temporal mobility of those people displaced internally during the immediate entering troops into their area and backed immediately to their homes after the occupation. This study also excluded the western zone of Tigray because it was occupied by perpetrator forces during the data collection period.

### Sampling and sampling technique

The sample size was determined based on the following assumptions. The study considered the enumeration areas of the Ethiopian Demographic and Health Survey (EDHS). About 48 woredas were used for enumeration. Each woreda had 4 enumeration sites each with 20 households (HHs) to be sampled per enumeration site. The required sample size was calculated as 48 woredas * 4 enumeration site/woreda * 20 HHs/enumeration = 3840 HHs. The final sample size was 3840 HHs.

A multi-stage sampling technique was used. All zones in Tigray were selected except the western zone and then 48 woredas were randomly selected and a systematic random sampling was employed to select HHs from the selected enumeration sites. The list/sampling frame of households that hosted IDPs was taken from the social affairs of the districts and towns. The smallest study unit of this survey was systematically selected households. We interviewed both the family heads of the displaced and host households. When there were 2 or more displaced families in one household, one was selected using simple random sampling. However, if the families came from different areas and if the one displaced is single or with no family and the other displaced is with his/her family, the displaced family (the one displaced with family) was selected purposively. If the displaced family backed to their home, questions related to the displaced were asked for the host household's head on behalf of the displaced one.

### Data collection tool and technique

The tool was developed by reviewing different kinds of literature and it has different sections such as socio-demographic characteristics, economic-related questions, and immediate health-related situations including the mental illness questions of the hosting households and displaced people were included on the tool. Data were collected using a structured and pretested questionnaire using complete and paper-based interviews of data collection techniques at the household level from all eligible households by trained data collectors and supervisors.

### Data quality assurance and management

The questionnaire was first prepared in English and translated to the local language Tigrinya language and then it was again translated back to English. The one-day training was given to data collectors and supervisors about detail items of the questionnaire and field guide. One week before the actual data collection, a pretest was done from a similar population outside the study area, and based on the findings of the pretest, minor modifications of questions, wordings, phrases, and time required to interview respondents were made. Each questionnaire collected from the field was checked for completeness, missed values, and unlikely responses and then manually cleaned up on such indications. Then data were coded and entered into a computer using Epi-data version 3.7 for entry customizing and skip benefit, then after data cleaning, it was exported to SPSS version 26 computer software packages. Data were cross-checked for consistency and accuracy.

### Data processing and analysis

After being transferred to SPSS we cleaned the data and prepared for analysis. Descriptions of the main findings were done using frequencies, percentages, and summary statistics. The descriptive analysis was made regarding characteristics classification using different tables and figures; determining the proportion and rates of each indicator. We used ArcGIS software to look at the burden of the host community by displaying the map distribution. The economic measurements were estimated by the cost that was recorded during the data collection period for all materials and assets destroyed or/and looted.

### Operational definition and measurements

#### Internally displaced persons (IDPs)

Persons or groups of persons who have been forced or obliged to flee or to leave their homes or places of habitual residence, in particular as a result of or to avoid the effects of armed conflict, situations of generalized violence, violations of human rights or natural or human-made disasters, and who have not crossed an internationally recognized State border^23^.

#### Community-hosted internally displaced persons (cIDPs)

Those IDPs displaced due to the war not living in central IDP camps but hosted in each household of the community.

#### Hosting households

Those households (HHs) permanently lived in the community and hosted IDPs in their home.

*Woreda*: is the equivalent name for the district.

*Tabia*: is the lowest administrative unit in a woreda.

### Ethics approval and consent to participate

The study was approved by the Institutional Ethical Review Board of Mekelle University, College of Health Science with IRB Ref: MU-IRB 1907/2021. Respondents were informed about the purpose of the study and oral informed consent was obtained from the study participants and their legal guardians to maintain confidentiality which is approved by the ethics committee/Institutional Review Board of Mekelle University, College of Health Science with IRB Ref: MU-IRB 1907/2021. Respondents were informed of their right to withdraw from the study at any time with no subsequent harm for refusal of participation. All methods were carried out per relevant guidelines and regulations or the declaration of Helsinki.

## Results

### Socio-demographic characteristics of the community hosted IDPs

The response rate was 3572 (93%) out of the 3840 households. The total IDPs in these HHs were 12,691. The average family size among the IDPS was six with a range of 1–11 members. About 44% of cIDPs stayed for 6 months and above in the hosting households. Though people from all parts of Tigray have been displaced, the number varies from place to place. Nearly 50% of the cIDPs were from the western and northwestern zones of Tigray. Mekelle the capital city consisted of only 06% (six) of the cIDPs contributed. The mean age of cIDPs was 36.2 (SD ± 12.12). About 51% of HH heads of cIDPs were females.

Regarding the education of the cIDPs, about 1/3rd had secondary education and 17.2% of heads of cIDPs had college and above. Thirty-two percent of the cIDPs respondents were farmers followed by 27.1% of merchants (Tables 1, 2).

**Table 1:** Socio-demographic characteristics of the Community hosted IDPs of Tigray, 2021 (N = 3572).

Variable	Classification	Frequency (percent)
Origin of displaced people (Zones of Tigray)	Southern	275 (8.0)
	Southeastern	243 (7.0)
	Mekelle	207 (6.0)
	Eastern	612 (17.7)
	Central	374 (10.8)
	Northwestern	684 (19.8)
	Western	1057 (30.6)
Sex	Male	1753
	Female	1825
Age (years)	< 20	158 (4.6)
	21–35	1806 (52.1)
	36–50	119 (32.3)
	> 50	383 (11.1)
Occupation	Farmers	1157 (32)
	Gov and non-gov employee	783 (22.7)
	Merchant and investor	972 (28.1)
	Daily laborer	542 (15.7)
Education	No formal education	351 (12.6)
	Primary	1016 (36.4)
	Secondary	946 (33.9)
	College and above	481 (17.2)
Family size	1–3	1493 (47.4
	4–6	1321 (41.9
	> 6	337 (10.7)

**Table 2:** Type of perpetrators and their duration of stay in the community of Tigray, 2021 (N = 3572).

Variable	Classification	Frequency (percent)
Time of stay as cIDPs	< 4 months	749 (21.7)
	4–6 months	551 (15.9)
	> 6 months	2157 (62.4)
Assets of cIDPs	Normal	411 (13.8)
	destroyed/burnt	509 (17.1)
	Looted	2053 (69.1)
Perpetrator force in the local area	Ethiopia troops	2091 (61.25)
	Eritrea troops	2206 (64.62)
	Amhara forces	1378 (40.37)
	Aliens of the three forces	679 (19.89)

### Who made cIDPs displaced from their home?

Of the total study participants, 64.6% of them claimed that they were displaced by Eritrea troops, 61.25% were displaced by Ethiopian troops and 40.4% were displaced by Amhara forces. In addition to this, 19.9% of cIDPs also claimed that they were displaced by the triple allied forces (Ethiopia troops + Eritrea troops + Amhara forces).

### Immediate health impact of the war on community IDPs

This study assessed the immediate health impact among internally displaced persons (IDPs) since the start of the war in Tigray. Approximately 536 (15%) IDPs reported that members of their families were killed by the invading forces. Out of these, 56.8% were killed by Eritrean forces. Physical disability caused by gunshot was reported by 244 (6.83%) of IDPs, and 439 (12.3%) who had chronic diseases with ongoing treatment reported that they had to stop taking their medication due to the war. Additionally, there were 636 (17.8%) IDPs suffering from various illnesses other than chronic diseases from frequent episodes of coughing up to one individual faced to deafness. Furthermore, 57.9% of IDPs reported a lack of access to medicines.

**Figure 1:**
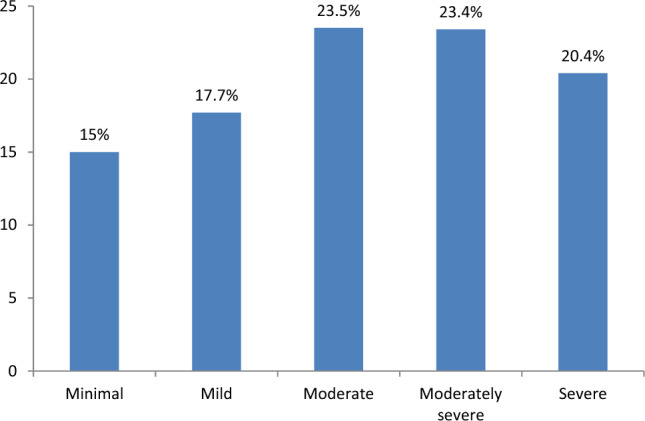
Severity of depression among the internally displaced persons hosted in the communities in Tigray, 2021.

Regarding the mental illness assessment, 43.8% and 58.8% of respondents’ cIDPs were suffering from severe depression and post-traumatic stress disorder (PTSD) respectively. About 294 (15.0%), 48 (17.7%), 23.5% (461), 23.4% (459), and 20.4% (400) of the IDPs had minimal, mild, moderate, moderately severe, and severe depression, respectively (Fig. 1).

### The immediate economic impact of the war on the community hosted IDPs

Among cIDPs claimed that 69.1% claimed that their household assets were looted and 15.8% reported it was destroyed (damaged or burnt) totally. The respondents claimed 76.2% of the looting and destruction was carried out by Eritrea troops, 66.0% by Ethiopian troops, and 60% by Amhara forces.

From the 3572 households of cIDPs, 22,713 domestic animals, 45,648 quintal cereals, machinery, cars, commercial and business materials, and house properties were looted by the perpetrator forces (Table 3).

**Table 3:** Economic impact reported by cIDPs due to the war in Tigray, 2021 (N = 3572).

Type	Quantity (observed)	Estimated (1.85 million IDPs)	Cost (Birr)	Estimated (to 1.85 million IDPs)
Domestic animals				
Cattle	4475	210,325	98,595,000.00	8,528,467,500.00
Sheep and Goat	11,838	556,386	40,168,400.00	3,474,566,600.00
Donkey, Mule and Horse	483	22,701	4,713,000.00	407,674,500.00
Chicken	5917	278,099	2,366,800.00	204,728,200.00
Cereals				
Cereals	45,648 quintal	2,145,456 quintal	208,954,000.00	18,074,521,000.00
Machinery and household properties				
Car	334	15,698	501,000,000.00	43,336,500,000.00
Television	1104	51,888	11,040,000.00	954,960,000.00
Refrigerator	711	33,417	14,220,000.00	1,230,030,000.00
Generator	219	10,293	5,475,000.00	473,587,500.00
Solar	576	27,072	2,880,000.00	249,120,000.00
Other properties*	….	….	669,000,000.00	57,868,500,000.00
Total			**1,558,412,200.00**	**134,803,000,000.00**

*Jewelry (gold), bed, sponges, money, phones, clothes, and other house properties.

Significant values are in [bold].

After the displacement, the main challenges of the cIDPs were assessed. From the total respondents, 86.6% lack clothes to wear, 80.4% lack a sleeping room, 88.1% experienced food shortage, and 77.0% lack blankets.

### The impact of the displacement on hosting households

The age of the respondents (host household) ranged from 17 to 89 years with a mean ± SD of 48.4 ± 14.5 years old. Of the total respondents, 62.5%, 11.2%, 10.2%, 5.7, and 9.7% were farmers, merchants, governmental and non-governmental workers, daily laborers, and with no job individuals respectively. 819 (30%) had no formal education and 245 (7%) of the respondents had an education in college and above. 2525 (74.4%) and 400 (11.8%) were married and widowed of their marital status. Regarding the family size of host households, 1743 (54.3%) and 1257 (39.2%) had a family of 5–8 and 1–4 respectively. 97.1% (3289) of host households were Orthodox in religion and 67 (2%) and 24 (0.7%) were Muslim and Catholic in religion respectively.

Before erupted the war, the family size of the hosting HHs was six with a range of 1–13, but after adding an average of four cIDPs (range of 1–12), the family size of the hosting HHs increased to 8 family sizes (range of 3–22). More than 37% of the HHs hosted more than 5 cIDPs, and about 43.4% of cIDPs were hosted for more than 6 months at the same HH.

From the 3576 hosting HHs, 11,303 domestic animals, 1629 quintal cereals,98 cars, 634 televisions, 226 different sizes of refrigerators, 140 generators, 501 solar, and other businesses and household properties were looted by the perpetrator forces. From 1420 HHs, the cost of the assets looted by the three forces was estimated up to 369,532,134.00 Birr (Table 4).

**Table 4:** Assets looted from the households hosting internally displaced people in Tigray, 2021 (N = 3572).

Type	Quantity	Cost (Birr)
Domestic Animals		
Cattle	765	50,328,400.00
Sheep and Goat	4058	8,718,100.00
Donkey, Mule and Horse	351	4,162,600.00
Chicken	6129	2,142,457.00
Cereals		
Cereals	1629 quintal	5,702,242.00
Machinery and household properties		
Car	98	147,000,000.00
Television	634	40,484,480.00
Refrigerator	226	3,586,300.00
Generator	140	2,750,500.00
Solar	501	2,505,000.00
Other properties*	….	102,152,055.00
Total		**369,532,134.00**

*Jewelry (gold), bed, sponges, money, phones, clothes, and other house properties.

Significant values are in [bold].

## Discussion

Based on this study done on 3,572 cIDPs, family members of cIDPs were killed (15% of cIDPs reported); the cIDPs were faced with illness, diseases and disability, discontinuation of medication, and health care follow-up and suffered psychological crises like depression and PTSD. There was also huge economic damage and distraction in both the hosting households and cIDPs such as destruction and looting of permanent assets, looting of domestic animals, food cereals, cars, different types of machinery, house commodities and materials, and civilian cash.

The war had a significant impact on the health of cIDPs. For instance, about 12% of cIDPs were suffering from chronic diseases and with follow-up medication were forced to stop their medication. 636 (17.8%) of cIDPs also suffered from different illnesses from frequent episodes of coughing up to one individual faced deafness. This is due to the deliberate distraction and stopping of the health care service in Tigray by the perpetrator forces^19^. Based on Medicine Sun Frontiers (MSF) report, and other studies at different times more than 80% of the health facilities of Tigray are destroyed by Ethiopian government troops, Eritrea government troops, and Amhara regional forces^18,19^. This resulted from the total collapsed health system of Tigray and the society of Tigray couldn't get health care service since the onset of the war^24^. Currently, there is not a single health facility fully functioning, in addition to the destruction the health system suffers from a severe shortage of human resources, medications, and other basic medical supplies^25^. As the WHO and other NGOs reported, malnutrition impacts a large portion of the population, and maternal and child health is disproportionally affected. Prevention and/or controlling COVID-19 and other epidemics are unthinkable. Even more worryingly, the collapsed health system has also left the nation vulnerable to any type of health problem and exacerbates the humanitarian situation^9–11,20,26–29^. From 3572 HH IDP participants, 536 (15%) civilian family members of cIDPs were killed (with a range of 1 up to 8 family members) at their home by the three forces. 244 (6.83%) of civilian family members faced physical disability from reversible gun wounds up to permanent disability. Consequentially, 43.8% and 58.8% of respondents of cIDPs suffered from severe depression and post-traumatic stress disorder (PTSD). This shows that, the severity and coverage of the health problem and that every other cIDPs has PTSD which is a very serious mental health crisis at the societal level.

Regarding the immediate economic impact of the war on cIDPs, from the 3576 households of cIDPs, 22,713 domestic animals, 45,648 quintal cereals, a lot of machinery, cars, commercial and business materials, and house properties were looted by the perpetrator forces. The respondents reported that 69.1% of cIDP's asset is looted and 15.8% is destroyed (damaged or burnt) totally. This shows that the severity of economic damage among the cIDPs was catastrophic. Based on different sources like Amnesty International, the destruction and looting action was intentionally used as a weapon of the war. As per the report of Assembly International and Human Rights Watch the intentional action taken by Eritrea and Amhara forces is very severe among the residents of western Tigray^20,21^. The perpetrators used looting and distraction as a weapon of the war and this resulted now very catastrophic humanitarian crises like man-made famine and malnutrition across the nation of Tigray^9,10,14,17,24,25,29–31^.

In addition to the cIDPs, the host households also faced huge economic damage due to the intentional looting and distraction of the perpetrators. From the 3576 hosting HHs, 11,303 domestic animals, 1629 quintal cereals, 98 cars, 634 televisions, 226 different sizes of refrigerators, 140 generators, 501 solar, and other Businesses and household properties were looted by the perpetrator forces. In addition to this, every household hosted eight cIDPs on average in his/her house for more than six months and above. This made holistic humanitarian crises in Tigray and all individuals need humanitarian help. The public basic structures such as banking, telecom, electricity, transportation, internet, and other basic infrastructures are blocked out in Tigray by the government of Ethiopia and this deteriorated the humanitarian crises. As the WHO director said, the blockage in Tigray is the longest siege in world history for more than 18 months since the onset of the war.

This study is done only in six zones of Tigray and didn’t include the western Tigray, and this may underestimate the problem. In addition to this, the study is done in the middle of the war, which means the severity of the situation may increase, and then the result of this study is represented only for its study period.

## Strength and limitation of the study

This study was done on IDPs, those hosted inside the community, not in IDP camps, means it assessed the neglected displaced individuals in most reports and organizations. The study used large sample size and helps on the representation of the findings. However, this study covered only the impact of the first eight months of the war (phase-1), but not included the next 16 months of the war. So that it doesn’t represent the whole impact of the war. Second, western Tigray was not included in this study due to that was under the perpetrators, and this may under-estimate the impact of the war and couldn’t show the whole figure. Finally, the information collected about amount and kind of looted assets of the IDPs was based on the IDPs report and this may expose to subjectivity of the evidence on the estimation of the impact.

## Conclusion

The war had a huge impact on the health and economy of the cIDPs and their hosting community. Illness, diseases and disability, discontinuation of medication and health care follow-up, and were suffered to psychological crises like depression and PTSD. There was also huge economic damage and distraction in both the hosting households and cIDPs such as destruction and looting of permanent assets, looting of domestic animals, food cereals, cars, different types of machinery, house commodities and materials, and civilian cash.

## Data Availability

All the data supporting the findings is contained within the manuscript, when there is in need the data set used for the present study's conclusion can be accessible from the corresponding author upon reasonable request.

## Abbreviations

CSA: Central Statistical Agency
EDHS: Ethiopian Demographic Health Survey
IDP: Internal Displaced Persons
cIDPs: Community hosted Internal Displaced Persons
HHs: Households
IDMC: Internal Displacement Monitoring Center
UNHCR: United Nations Higher Commissioner Refuges
TDF: Tigray Defense Force
GIS: Geographic Information System

## References

[1] Levy BS, Sidel VW (2008). War and Public Health.

[2] World Health Organization (2011). War Trauma Foundation, World Vision International. Psychological first aid: Guide for field workers.

[3] Gray B, Hanna F, Reifels L (2020). The integration of mental health and psychosocial support and disaster risk reduction: A mapping and review. Int. J. Environ. Res. Public Health.

[4] Yigzaw GS, Abitew EB (2019). Causes and impacts of internal displacement in Ethiopia. Afr. J. Soc. Work..

[5] The US government and internally displaced persons: Present, but not accounted for. Brookings Institution and the US Committee for Refugees; 1999 Nov (Mooney, E. The concept of internal displacement and the case for internally displaced persons as a category of concern. *Refug. Surv. Q.* **24**(3), 9–26 (2005).

[6] UN High Commissioner for Refugees (UNHCR). Internally Displaced People. Questions & Answers, September 2007, UNHCR/MRPI/Q&A A•3/ENG 1. https://www.refworld.org/docid/47a7078e1.html. Accessed 28 Apr 2023.

[7] Leus X, Wallace J, Loretti A (2001). Internally displaced persons. Prehosp. Disaster Med..

[8] UN High Commissioner for Refugees (UNHCR), Internally Displaced People. Questions & Answers, September (2007).

[9] EHRC/OHCHR. Report of the Ethiopian Human Rights Commission (EHRC)/Office of the United Nations High Commissioner for Human Rights (OHCHR) Joint Investigation into Alleged Violations of International Human Rights, Humanitarian, and Refugee Law Committed by all Parties to the Conflict in the Tigray Region of the Federal Democratic Republic of Ethiopia.

[10] OCHA. Ethiopia—Tigra y region humanity Arian update Situation Report Last updated: 14 Jan 2021 (2021).

[11] OCHA. Humanitarian response plan monitoring report: Humanitarian programme cycle January–June 2021 (2021).

[12] Roberta C (2012). The guiding principles on internal displacement: A new instrument for international organizations and NGOs. Forced Migr. Rev..

[13] IDMC and NRC. Global Report on Internal Displacement (2022).

[14] USAID. Ethiopia—Tigray Crisis September 30, 2021 (2021).

[15] Araya, M. Postconflict internally displaced persons in Ethiopia: Mental distress and quality of life about traumatic life events, coping strategy, social support, and living conditions [Internet] [Ph.D. dissertation]. [Umeå]: Psykiatri; 2007. (Umeå University medical dissertations). http://urn.kb.se/resolve?urn=urn:nbn:se:umu:diva-1434

[16] Richards A (2011). Posttraumatic stress disorder, anxiety and depression symptoms, and psychosocial treatment need in Colombians internally displaced by armed conflict: A mixed-method evaluation. Psychol. Trauma Theory Res. Pract. Policy.

[17] World Vision. Assessment of internally displaced people in Tigray. January (2021).

[18] Gesesew H, Berhane K, Siraj ES, Siraj D, Gebregziabher M, Gebre YG (2021). The impact of war on the health system of the Tigray region in Ethiopia: An assessment. BMJ Glob. Health.

[19] MSF. People were left with few healthcare options in Tigray as facilities were looted, and destroyed: Médecins sans Frontières (MSF). https://www.MSF.org/health-facilities-targeted-tigray-region-Ethiopia (2021).

[20] Human Rights Watch, and Amnesty International. We will erase you from this land” crimes against humanity and ethnic cleansing in Ethiopia’s Western Tigray Zone (2022).

[21] Amnesty International. We will erase you from this land” crimes against humanity and ethnic cleansing in Ethiopia’s Western Tigray Zone. April (2022).

[22] Lawrence KC (2021). A study on the psycho-social factors associated with the mental health of uniformed personnel in internally displaced person camps in Nigeria. Commun. Ment. Health J..

[23] Global Protection Cluster Working Group. Handbook for the protection of internally displaced persons. (No Title) (2007).

[24] Tesfay F, Gesesew H. The health crisis in Ethiopia’s war-ravaged Tigray. Ethiopia Insight (2021).

[25] D’Costa B (2022). Tigray’s complex emergency, expulsions and the aspirations of the responsibility to protect. Glob. Responsib. Prot..

[26] UNHCR. Ethiopia situation (Tigray region) 1 March–15 March (2021).

[27] UNHCR. UNHCR situation update Ethiopia, Tigray 30 July 2021 (2021.

[28] UNHCR. UNHCR ethiopia operation: Tigray situation update, 06 September 2021.

[29] Ghimire M. Ethiopia's Internet Shutdowns: Contributing to the humanitarian catastrophe in the Tigray.

[30] Kesto DA, Tamene H (2022). Emergency Project Management Practice in Ethiopia: The case of world vision humanitarian crises response projects in Tigray. Int. J. Project Manag. Product. Assess. (IJPMPA)..

[31] OCHA. Ethiopia—Northern Ethiopia humanitarian update situation report, 3 Mar 2022.

